# Opportunities for change and levelling up: a trust wide retrospective analysis of 8 years of laparoscopic and abdominal myomectomy

**DOI:** 10.52054/FVVO.16.2.025

**Published:** 2024-06-28

**Authors:** N.A.M. Cooper, N.F. Daniels, Z Magama, M Aref-Adib, F Odejinmi

**Affiliations:** Whipps Cross University Hospital, Barts Health NHS Trust, London, E11 1NR UK; Women’s Health Research Unit, Wolfson Institute of Population Health, Queen Mary University London, London, E1 2AB UK

**Keywords:** Fibroids, myomectomy, laparoscopy, cost, minimally invasive surgery

## Abstract

**Background:**

Laparoscopic myomectomy is increasingly considered the gold standard uterine preserving procedure and has well documented benefits over the open approach. Barriers that women have in accessing the most appropriate treatment need to be addressed to ensure optimal patient care and outcomes.

**Objectives:**

To analyse rates of open and laparoscopic myomectomy at a large NHS trust and identify how many cases could potentially have been performed laparoscopically, and any variation between sites.

**Material and Methods:**

A retrospective review of preoperative imaging reports and a surgical database containing information for all myomectomies performed between 1st January 2015 and 31st December 2022.

**Main outcome measures:**

Number of procedures suitable for alternative surgical approach; length of hospital stay; estimated blood loss; cost differences.

**Results:**

846 myomectomies were performed; 656 by laparotomy and 190 by laparoscopy. 194/591 (32.8%) open myomectomies could have been performed laparoscopically and 26/172 (15.1%) laparoscopic myomectomies may have been better performed via an open approach. Length of hospital stay, and estimated blood loss were significantly higher in the open group. Had cases been performed as indicated by pre-operative imaging, the cost differences ranged from -£115,752 to £251,832.

**Conclusions:**

There is disparity in access to the gold standard care of laparoscopic myomectomy. Due to multifactorial reasons, even at sites where the rate of laparoscopic myomectomy is high, there is still underutilisation of this approach. It is clear that there is scope for change and “levelling up” of this imbalance.

**What is new?:**

Robust pathways and guidelines must be developed, and more laparoscopic surgeons should be trained to optimise care for women with fibroids.

## Introduction

Fibroids are the most common benign tumour of the female genital tract. The true prevalence remains unknown as many women with fibroids are asymptomatic (Laughlin et al., 2010). Reports suggest that they are present in up to 70% of women before the menopause (Stewart et al., 2017) For women who do have symptoms, the effect on their daily lives can be devastating, leading to loss of intimate relationships, subfertility, and pregnancy loss. The most common symptoms of heavy periods and pressure cannot be overlooked as they cause chronic restrictions on every aspect of women’s lives (Cooper et al., 2023). Fibroids are more commonly diagnosed in Black and Asian ethnic minority groups (Baird et al., 2003) and these groups often suffer from disparity in access to care (Sengoba et al., 2017). Poorer post- operative outcomes have been reported in this group of women compared to Caucasian, despite the same fibroid burden (Stentz et al., 2018).

There are many treatment options for fibroids however, hormones are not suitable for women who want immediate fertility, uterine artery embolisation is not recommended for women seeking pregnancy and abdominal myomectomy increases the risk of adhesions. For women with large fibroids wishing to achieve a pregnancy or preserve their uterus, the optimal option is myomectomy, preferably laparoscopic or robotic. Laparoscopic myomectomy (LM) is increasingly considered the gold standard uterine preserving procedure in carefully selected women (Mallick and Odejinmi, 2017) and this minimally invasive approach has well-documented benefits over the open approach including quicker recovery and return to normal activity, reduced blood loss, and reduced post-operative pain (Chittawar et al., 2014). However, the controversy surrounding intra- abdominal morcellation (Odejinmi et al., 2019) and constraints around the size and number of fibroids that can be tackled laparoscopically (Sinha et al., 2008) mean that it is not suitable for all women (Catanese et al., 2022). A final consideration is that LM requires advanced minimally invasive surgical techniques that require specialist training, the availability of which varies between regions and hospitals across the United Kingdom (UK) (Aref- Adib et al., 2023).

Barts Health NHS Trust is the second largest in the UK, serving a diverse population of approximately 2.5 million people. Services are provided in over 40 languages across four main hospital sites. The primary aim of this study was to analyse the rates of open and laparoscopic myomectomy in this trust and identify how many open cases could potentially have been performed laparoscopically. Secondly, we assessed whether variation existed with regard to access to LM between the different sites within the trust. In addition, length of hospital stay, estimated blood loss, and potential financial implications were explored.

## Methods

A retrospective review of a surgical database containing information for all myomectomies performed at Barts Health NHS trust between 1st January 2015 and 31st December 2022 was undertaken. An integrated research application was undertaken, however as this was deemed to be an audit of existing practice, formal ethical approval was not required, and the audit was registered with Barts Health Clinical Effectiveness Unit (project number 12980). All transcervical, vaginal myomectomies and those performed at the time of caesarean section were excluded, as the criteria for these procedures differ from those for elective abdominal myomectomy. Data analysed included patient demographic data, intra-operative and post-operative details. Though laparoscopic myomectomy depends on the skill and expertise of the surgeon, minimum standards were set using preoperative imaging reports. There is no standardised protocol for scan reports within the trust (and this did not change over the period of the study) however reports included the number and size of the fibroids. The scan reports were used to identify which cases could have been suitable for a laparoscopic approach using these previously published criteria: i) no more than three fibroids, ii) the largest fibroid having a maximum diameter of 10cm (still applicable with a solitary fibroid) (Nezhat et al., 1996; Dubuisson and Chapron, 1996), as most laparoscopic surgeons would be able to manage such cases laparoscopically. This way we identified which of the open myomectomies performed had the potential to have been done laparoscopically and laparoscopic myomectomies which may have been more suitable for open myomectomy, particularly as emerging data demonstrate an increased risk of recurrence and adhesion formation with an increasing number of fibroids removed at LM (Ming et al., 2020; Bortoletto et al., 2022). In addition, the estimated blood loss and length of stay for each type of procedure were compared. When estimated blood loss was reported as ‘minimal’ it was replaced by a value of 50 millilitres. Statistical analyses were performed using IBM SPSS Statistics version 24. Groups were then compared using a Students-t test. Length of stay data was analysed using the Mann-Whitney-U test. Data were examined as a whole and by individual sites within the trust.

Healthcare Resource Group (HRG) codes, which are used in the NHS to set tariffs for procedures, were used for cost calculations.

## Results

Between 1st January 2015 and 31st December 2022, 846 abdominal myomectomies were performed across Barts Health NHS trust. 656 of these procedures were performed by laparotomy and 190 by laparoscopy. Table I shows the breakdown of these procedures by hospital site. As a trust, 22.5% of myomectomies were performed via laparoscopy, however, this varied across sites, ranging from 0% to 50.2%. In 2015 the proportion of myomectomies being done by a laparoscopic approach was 21% and in 2019 this had risen to 28%. However, following the global COVID-19 pandemic, in 2022 rates had fallen to 17%.

**Table I:** Abdominal Myomectomies across Barts Health NHS trust 2015-2022.

Hospital site	Open myomectomy	Laparoscopic myomectomy	Total	% Laparoscopic
Site 1	300	14	314	4.5%
Site 2	118	119	237	50.2%
Site 3	236	57	293	19.5%
Site 4	2	0	2	0.0%
Trust wide	656	190	846	22.5%

The mean age was 38.89 (SD 5.96) years in the open myomectomy group and 37.47 (SD 6.23) in the laparoscopic myomectomy group. A total of 26 women were aged 50 years or above: 5 in the laparoscopic group and 21 in the open group.

48.7% women were of Black ethnic origin, 18.4% Asian, 13.1% white, 5.9% mixed, Chinese or other, and 13.8% unknown.

Pre-operative imaging reports were available for 763/846 (90.1%) cases; 591/656 (90.0%) open cases and 172/190 (90.5%) laparoscopic cases. When applying the prespecified criteria to cases with preoperative imaging, 397/591 abdominal myomectomies were appropriately performed via an open approach, however, 194/591 (32.8%) could have been performed laparoscopically. When explored by site, the proportions that could have been performed laparoscopically ranged from 25.8% to 35%, except for a single site that only performed two myomectomies over the time frame, both of which were done via laparotomy, despite both being suitable for laparoscopy. See Table II.

**Table II:** A table showing the numbers of women receiving myomectomy via the indicated surgical approach or via an alternative approach.

Site	Number of cases	Cases with scan data	Number undergoing the appropriate surgical approach	Number who could have had the alternative surgical approach
Open myomectomy				
Site 1	300	260	169	91 (35.0%)
Site 2	118	97	72	25 (25.8%)
Site 3	236	232	156	76 (32.8%)
Site 4	2	2	0	2 (100%)
Trust wide	656	591	397	194 (32.8%)
Laparoscopic myomectomy				
Site 1	14	13	12	1 (7.7%)
Site 2	119	106	85	21 (19.8%)
Site 3	57	53	49	4 (7.5%)
Site 4	0	0	0	0 (0%)
Trust wide	190	172	146	26 (15.1%)

When reviewing the laparoscopic myomectomies, 146/172 were appropriately performed laparoscopically but 26/172 (15.1%) did not meet (exceeded) the criteria for this surgical approach. Table II shows these data in more detail.

Ethnicity did not appear to be a factor with Black women having a 26% risk of getting an inappropriate procedure, compared to approximately 33% in all other ethnic groups.

A Mann-Whitney test indicated that the length of hospital stay was significantly higher in the open group when compared to the laparoscopy group (median 3 days vs 1 day, p<0.001).

Estimated blood loss data were available from documented records for 455/656 open cases and 158/190 laparoscopic cases. There was a significantly higher mean estimated blood loss in the open group 667ml (SD 780ml) versus the laparoscopic group 251ml (SD 281ml), mean Difference 416.51, p<0.001.

Data from the 763 patients with scan data available were utilised in the cost calculations. Three analyses were performed, the first used the healthcare resource group (HRG) code for ‘major’ versions of the operations, the second used the code for the ‘complex’ version, and the third analysis used ‘major for the open approach and ‘complex for the laparoscopic approach. The cost differences ranged from -£115,752 (major vs complex) to £251,832 (complex vs complex) as highlighted in Table III.

**Table III:** Estimates of cost differences between the procedures that happened versus what might have happened had all women had received the surgical approach indicated by the pre-operative imaging.

	Number of cases	Using ‘major’^*^ unit costs for both (£)	Using ‘complex’^^^ unit costs for both (£)	Using ‘complex’^^^ unit costs for laparoscopic and ‘major’^*^ for open (£)
ACTUAL				
Open myomectomy	591	2,894,127	4,187,235	2,894,127
Laparoscopic myomectomy	172	775,204	960,792	960,792
Total	763	3,669,331	5,148,027	3,854,919
EXPECTED				
Open myomectomy	423	2,071,431	2,996,955	2,071,431
Laparoscopic myomectomy	340	1,532,380	1,899,240	1,899,240
Total	763	3,603,811	4,896,195	3,970,671
COST DIFFERENCE (£)		65,520	251,832	-115,752

^*^ Major cost for open myomectomy= MA07G Major open upper genital tract Procedure with CC score 0-2 £4897; Major cost for laparoscopic myomectomy= MA08B Major, laparoscopic or endoscopic upper genital tract procedures with CC Score 0-1 £4507.

^^^Complex cost for open myomectomy= MA01Z Complex open upper or lower genital tract procedures £7085; Complex cost for laparoscopic myomectomy MA28Z Complex, Laparoscopic or Endoscopic, Upper Genital Tract Procedures £5586.

## Discussion

The minimal access approach to gynaecological surgery has well-documented advantages (Aarts et al., 2015) and this has resulted in increased numbers of laparoscopic hysterectomies worldwide (Lycke et al., 2021; Tyan et al., 2022). Despite this increase there remains a disparity in the outcome and access to laparoscopic hysterectomy within health care systems as demonstrated in an Australian cohort of women who underwent different procedures on different sites of a health care delivery system (Higgins et al., 2022). In some countries, the centralisation of services for the treatment of advanced endometriosis has been shown to improve outcomes for women with endometriosis (Bendifallah et al., 2018; Byrne et al., 2018). Globally there has also been an increase in the number of laparoscopic myomectomies (Dallas et al., 2021) but despite this, there are reports of disparity in access and outcomes for women with fibroids requiring minimal access procedures (Stentz et al., 2018). Our study presents evidence of disparity in access despite being a trust with relatively high LM rates.

In 2018-19, 2755 myomectomies were performed in England, with 48% happening in London. Only two hospital trusts in London (Imperial College Healthcare NHS Trust and Guy’s and St Thomas’ Foundation NHS Trust) performed more myomectomies than Barts Health and Barts health was the second largest provider of LM. Barts Health operates within the NHS where health care is free to all at the point of delivery and where everyone has equal opportunity to access care. All sites offer hysteroscopic myomectomy and have access to uterine artery embolisation and newer radiofrequency ablative techniques are being introduced. The four hospital sites that provide gynaecological care vary in that one is a tertiary unit providing mainly cancer and fertility treatment (site 4), whilst the other sites provide benign gynaecology services. Site 1 performed 4.5% of its myomectomies laparoscopically, whilst site 2 performed 50.2% via this route. It is apparent that the likelihood of a woman getting the option of LM may be impacted significantly by which hospital she attends as well as the surgical expertise available. With the largest distance between hospital sites being approximately 7 miles it seems unjust that women living a very short distance apart are not given the same opportunity to access the appropriate operation for them. Although there are reports in the literature of disparities with regards to access to care and treatment outcomes in the management of fibroids (Ptacek et al., 2021), ethnicity did not appear to be a factor that contributed to patients not being offered the surgical approach considered best for them in our study. We can hypothesise that this difference was due to available surgical expertise and referral pathways.

Despite Barts Health being a “highly performing institution” for LM compared with the national average (22.5% vs 18%), our data show that there is still room for increasing the rate. Despite there being a higher prevalence of laparoscopic myomectomy at Site 2, there were still 25 women (25.8% of the open myomectomy cases with scan data) who were potentially suitable for this procedure but underwent laparotomy. This was lower than at sites 1 and 3 with 91 (35.0%) and 76 (32.8%) women respectively. Thus, despite being the second highest provider of LM in London, the trust is still underusing this approach to myomectomy in potentially suitable women. There were a handful of patients who underwent a LM, who according to our criteria should have had their surgery via an open approach. 12/26 patients in this group had fibroids greater than 10cm in diameter and 14 had more than 3 fibroids. In this group of patients, the highest number of fibroids on pre-op imaging was six and the largest fibroid on pre-operative imaging had a diameter of 14.9cm. Though there is no reported increased risk of immediate complications in patients with large or increasing number of fibroids (Mallick and Odejinmi, 2017), LM in large or multiple fibroids could potentially increase the risk of adhesion formation (Bortoletto et al., 2022) or the risk of fibroid recurrence (Catanese et al., 2022). 19/26 patients were operated on by one surgeon (9 with fibroids >10cm and 10 with >3 fibroids) working in the hospital with the highest rate of LM, hence may reflect the confidence of that individual with performing the procedure. However, as referenced above the criteria we used for LM was the minimum standard, acknowledging that open myomectomy is still a procedure needed by some patients.

The criteria utilised in the study to assess suitability for a LM was modified from studies in the wider literature (Dubuisson and Chapron, 1996; Nezhat et al., 1996). Acknowledging that with technological advances as suggested by Sinha et al. (2008) more and larger fibroids should be suitable to remove via a laparoscopic approach.

Our data support previous findings that laparoscopic surgery is associated with lower blood loss and shorter length of stay when compared to laparotomy. With these and many more clear advantages of minimally invasive surgery over conventional open surgery there is a drive to increase the number of gynaecological procedures performed laparoscopically and this is clearly the trend being seen with laparoscopic hysterectomy rates (Madhvani et al., 2019). Despite this increase, barriers still exist to the provision of LM for suitable women.

The use of LM trust-wide increased from 21% in 2015 to 28% in 2019, in keeping with the national trend of increasing rates of LM due to more gynaecologists offering the procedure (Sirkeci et al., 2017). Rates of LM at Barts Health have been consistently above the national average of 18% (Amoah and Quinn, 2023) , although by 2022 rates at the trust had plummeted to 17.1% which is likely a reflection of the lengthy waiting lists created by the COVID-19 pandemic (Royal College of Obstetricians and Gynaecologists, 2022). Women have been forced to accept a more available yet inferior procedure rather than continue to wait. These low rates can be increased substantially to provide benefits to patients and the healthcare system. It is therefore imperative to identify barriers to the use of LM in suitable women who meet the criteria.

One barrier may be concerns regarding morcellation and sarcoma, particularly since the FDA blacklisting of electromechanical morcellation in the USA (FDA, 2014). These concerns remain despite the actual risk of a fibroid being sarcomatous of approximately 1:2000 in analysis by Pritts et al. (2015). Furthermore, there are other important issues to address with regards to morcellation (Odejinmi et al., 2019). As the risks associated with open surgery likely outweigh the risk of morcellation (Siedhoff et al., 2017), it is now accepted that morcellation should only be carried out inside a containment bag, not only to prevent the spread of sarcoma but also for benign disease e.g., parasitic fibroids (Venturella et al., 2016). Regardless, women need to be counselled on the risks.

Another barrier could be a lack of training. LM is a relatively technical, complex procedure when compared to other benign gynaecological surgery due to the amount and location of laparoscopic suturing required. Thus, it does not tend to be a procedure that all gynaecologists are able to offer. In fact, in the current Royal College of Obstetricians and Gynaecologists curriculum, UK trainees can complete their training without even observing LM being performed. Looking into the future, one argument would be that if we aren’t training surgeons to perform the procedure, we cannot expect it to be widely available. Thus, gaining expertise in uterine preserving fibroid surgery should be prioritised within the advanced surgical training modules. Of course, it is not only the surgeon’s ability to perform a LM that may limit it being offered. Even where surgeons are trained, the approach may be affected by the potential perceived difficulty; objective grading tools can be used to solve this problem (Leung et al., 2018). In order to improve access to minimal access myomectomy, strategies need to be put in place at both local and national levels to improve access to training. The introduction of Robotics has also been shown to increase the number of minimal access surgeries for women with greater fibroid burden (Jansen et al., 2019) and large fibroids (Lee et al., 2018).

The route of myomectomy was decided primarily by the clinician responsible for the patient. Getting it right first time (GIRFT) is a national scheme in the UK underpinned by the principle that the right patient should be operated on by the right surgeon at the right place and at the right time. The application of these principles should potentially help to increase the number of myomectomies performed laparoscopically. This however is dependent on the development of pathways and adequate training. The GIRFT scheme also advocates that trusts develop pathways to ensure this model of care. Pathways are dependent on evidence-based medicine and robust guidelines. Unfortunately, National guidelines for fibroids care are not entirely based on up-to-date evidence (Amoah et al., 2022), thus it is important to develop local and national care pathways for the holistic management of uterine fibroids. This will enable patients who are suitable for LM to be diverted to centres with the skills and infrastructure to perform the surgery. The aim of such pathways is not to compromise autonomy in clinical practice, but to develop a method of reducing disparity in access to care and to achieve better outcome for patients. Surgeons have the responsibility to discuss all options available for fibroid management and to offer referral to a colleague or another institution if they do not perform LM themselves as this is in the best interests of the patient.

Though LM would be more profitable for the health care system as a whole, our cost analysis is based on the use of HRG codes and the reimbursement costs for procedures rather than the actual cost of performing the procedures. The reimbursement fee may not accurately reflect the actual costs to the hospital, for example, does the reduced cost of a shorter hospital stay compensates for the use of expensive specialist laparoscopic instruments e.g., advanced bipolar, harmonic?

There are limitations to our study. Firstly, our data collection was retrospective and there was no data relating to patient preferences or patient desired outcomes. Neither was there data on surgeons’ attitudes towards LM, ability to perform the procedure, or attitudes towards morcellation. This would have given a clinical context of both input and output data to inform the relevance of the ratios of open versus laparoscopic myomectomy presented in our study and allow a more thematic analysis.

This study demonstrates that despite being in a well-performing health care system, there is still a disparity in access to the gold standard care of LM. Furthermore, even where there was an LM rate of 50.2%, there was still underutilisation of LM. Despite the limitations of this work, it is clear that there is scope for change and “levelling up” of this disparity in access to care.

More can be done to allow women to benefit from the advantages conferred by the minimally invasive approach to surgery for myomectomy if they wish to have it and meet criteria for the procedure, rather than be subjected to an open procedure just because it is the only one available. Departments and NHS trusts should ensure robust pathways are created and guidelines followed. There is also a place for patient education, so women know what is available and the inherent benefits of the various approaches to allow informed decision-making.

Nationally, the need to train laparoscopic surgeons to perform LM should be recognised, to ensure that like laparoscopic hysterectomy, it becomes the expected approach rather than an operation for the few who end up being in the right place at the right time.

## References

[1] Aarts JWM, Nieboer TE, Johnson N (2015). Surgical approach to hysterectomy for benign gynaecological disease.. Cochrane Database Syst Rev.

[2] Amoah A, Joseph N, Reap S (2022). Appraisal of national and international uterine fibroid management guidelines: a systematic review.. BJOG.

[3] Amoah A, Quinn SD (2023). Uterine-preserving treatments or hysterectomy reintervention after myomectomy or uterine artery embolization: a retrospective cohort study of long-term outcomes. BJOG.

[4] Aref-Adib M, Strong S, Ojukwu O (2023). Why and Where Are Interventions Performed: A Retrospective Analysis of Myomectomy for Uterine Fibroids in England (2018-2019).. J Minim Invas Gyn.

[5] Baird DD, Dunson DB, Hill MC (2003). High cumulative incidence of uterine leiomyoma in black and white women: ultrasound evidence.. Am J Obstet Gynecol.

[6] Bendifallah S, Roman H, Rubod C (2018). Impact of hospital and surgeon case volume on morbidity in colorectal endometriosis management: a plea to define criteria for expert centers.. Surg Endosc.

[7] Bortoletto P, Keefe KW, Unger E (2022). Incidence and risk factors of intrauterine adhesions following myomectomy.. F S Rep.

[8] Byrne D, Curnow T, Smith P (2018). Laparoscopic excision of deep rectovaginal endometriosis in BSGE endometriosis centres: a multicentre prospective cohort study.. BMJ Open.

[9] Catanese A, Siesto G, Cucinella G (2022). Factors influencing surgical outcomes of laparoscopic myomectomy. A propensity-score matched analysis. Prz Menopauzalny.

[10] Chittawar PB, Franik S, Pouwer AW (2014). Minimally invasive surgical techniques versus open myomectomy for uterine fibroids.. Cochrane Database Syst Rev.

[11] Cooper NAM, Yorke S, Tan A (2023). Qualitative study exploring which research outcomes best reflect women’s experiences of heavy menstrual bleeding: stakeholder involvement in development of a core outcome set.. BMJ Open.

[12] Dallas K, Molina AL, Siedhoff MT (2021). Myomectomy Trends in a population-based cohort from 2005-2018.. J Minim Invas Gyn.

[13] Dubuisson JB, Chapron C (1996). Laparoscopic myomectomy today: a good technique when correctly indicated.. Hum Reprod.

[14] (2014. Updated 2020). Laparoscopic Uterine Power Morcellation in Hysterectomy and Myomectomy: FDA Safety Communication.

[15] Higgins C, Mcdonald R, Mol BW (2022). Indications and surgical route for hysterectomy for benign disorders: a retrospective analysis in a large Australian tertiary hospital network.. Arch Gynecol Obstet.

[16] Jansen LJ, Clark NV, Dmello M (2019). Perioperative outcomes of myomectomy for extreme myoma burden: comparison of surgical approaches.. J Minim Invas Gyn.

[17] Laughlin SK, Schroeder JC, Baird DD (2010). New directions in the epidemiology of uterine fibroids.. Semin Reprod Med.

[18] Lee CY, Chen IH, Torng PL (2018). Robotic myomectomy for large uterine myomas.. Taiwan J Obstet Gynecol.

[19] Leung M, Murji A, Allaire C (2018). Factors influencing the difficulty of laparoscopic myomectomy: the development of a surgical rating tool.. Eur J Obstet Gynecol Reprod Biol.

[20] Lycke KD, Kahlert J, Damgaard R (2021). Trends in hysterectomy incidence rates during 2000–2015 in Denmark: shifting from abdominal to minimally invasive surgical procedures.. Clin Epidemiol.

[21] Madhvani K, Curnow T, Carpenter T (2019). Route of hysterectomy: a retrospective, cohort study in English NHS Hospitals from 2011 to 2017.. BJOG.

[22] Mallick R, Odejinmi F (2017). Pushing the boundaries of laparoscopic myomectomy: a comparative analysis of peri-operative outcomes in 323 women undergoing laparoscopic myomectomy in a tertiary referral centre.. Gynecol Surg.

[23] Ming X, Ran XT, Li N (2020). Risk of recurrence of uterine leiomyomas following laparoscopic myomectomy compared with open myomectomy.. Arch Gynecol Obstet.

[24] Nezhat F, Seidman DS, Nezhat C (1996). Hum Reprod. Laparoscopic myomectomy today: why, when and for whom.

[25] Odejinmi F, Aref-Adib M, Liou N (2019). In Vivo. Rethinking the Issue of Power Morcellation of Uterine Fibroids: Is Morcellation the Real Problem or Is this Another Symptom of Disparity in Healthcare Provision.

[26] Pritts EA, Vanness DJ, Berek JS (2015). The prevalence of occult leiomyosarcoma at surgery for presumed uterine fibroids: a meta-analysis.. Gynecol Surg.

[27] Ptacek I, Aref-Adib M, Mallick R (2021). Each Uterus Counts: A narrative review of health disparities in benign gynaecology and minimal access surgery.. Eur J Obstet Gynecol Reprod Biol.

[28] (2022). Left for too long: understanding the scale and impact of gynaecology waiting lists. https://www.rcog.org.uk/about-us/campaigning-and-opinions/left-for-too-long-understanding-the-scale-and-impact-of-gynaecology-waiting-lists/.

[29] Sengoba KS, Ghant MS, Okeigwe I (2017). Racial/Ethnic Differences in Women’s Experiences with Symptomatic Uterine Fibroids: a Qualitative Assessment.. J Racial Ethn Health Disparities.

[30] Siedhoff MT, Doll KM, Clarke-Pearson DL (2017). Laparoscopic hysterectomy with morcellation vs abdominal hysterectomy for presumed fibroids: an updated decision analysis following the 2014 Food and Drug Administration safety communications. Am J Obstet Gynecol.

[31] Sinha R, Hegde A, Mahajan C (2008). J Minim Invasive Gynecol. Laparoscopic myomectomy: do size, number, and location of the myomas form limiting factors for laparoscopic myomectomy.

[32] Sirkeci RF, Belli AM, Manyonda IT (2017). Treating symptomatic uterine fibroids with myomectomy: current practice and views of UK consultants.. Gynecol Surg.

[33] Stentz NC, Cooney LG, Sammel MD (2018). Association of Patient Race With Surgical Practice and Perioperative Morbidity After Myomectomy.. Obstet Gynecol.

[34] Stewart EA, Cookson CL, Gandolfo RA (2017). Epidemiology of uterine fibroids: a systematic review.. BJOG.

[35] Tyan P, Hawa N, Carey E (2022). Trends and perioperative outcomes across elective benign hysterectomy procedures from the ACS-NSQIP 2007–2017. J Minim Invasive Gynecol.

[36] Venturella R, Rocca ML, Lico D (2016). In-bag manual versus uncontained power morcellation for laparoscopic myomectomy: randomized controlled trial.. Fertil Steril.

